# Promastigote-to-Amastigote Conversion in *Leishmania* spp.—A Molecular View

**DOI:** 10.3390/pathogens11091052

**Published:** 2022-09-15

**Authors:** Joachim Clos, Janne Grünebast, Myrine Holm

**Affiliations:** Leishmania Genetics Group, Bernhard Nocht Institute for Tropical Medicine, D-20359 Hamburg, Germany

**Keywords:** post-transcriptional gene regulation, heat shock proteins, cell stress, protein kinases, protein turnover

## Abstract

A key factor in the successful infection of a mammalian host by *Leishmania* parasites is their conversion from extracellular motile promastigotes into intracellular amastigotes. We discuss the physical and chemical triggers that induce this conversion and the accompanying changes at the molecular level crucial for the survival of these intracellular parasites. Special emphasis is given to the reliance of these trypanosomatids on the post-transcriptional regulation of gene expression but also to the role played by protein kinases, chaperone proteins and proteolytic enzymes. Lastly, we offer a model to integrate the transduction of different stress signals for the induction of stage conversion.

## 1. *Leishmania* Biology and Life Cycle

### 1.1. Natural Life Cycle

All *Leishmania* parasites undergo a life cycle involving two different hosts, namely sand fly vectors (Diptera) and susceptible mammals, mostly rodents but also dogs and humans. To adapt and survive in their hosts, the leishmaniae exist in various flagellated forms inside the digestive tract of sand flies and as slowly proliferating or quiescent, non-motile amastigotes within macrophages and other mononuclear phagocytes.

*Leishmania* spp. are taken up by female flies into their digestive tracts as part of the blood meal. Within the peritrophic matrix, the parasites convert into flagellated promastigotes, which attach to the midgut epithelium via *Leishmania* surface molecules, e.g., lipophosphoglycan. These procyclic promastigotes proliferate logarithmically until they reach a stationary phase and undergo differentiation into small actively motile forms, the metacyclic promastigotes. The latter detach from the midgut epithelium and infect another mammal during the sand fly’s next bloodmeal [1,2]. Recent data also suggest that the *Leishmania* (*Mundinia*) subgenus may be transmitted by midges (genus *Culicoides*) [3]. Procyclic promastigotes of most *Leishmania* species can be generated in tissue culture media.

In mouse models, the injected parasites attract neutrophil granulocytes and tissue macrophages [4]. Inside these cells, leishmaniae convert into non-motile amastigotes, which persist in phagosomes, averting anti-microbial mechanisms by exporting effector molecules, i.e., regulatory proteins. They interfere with the signal transduction pathways of the host cells, modulating the host’s immune response [5].

### 1.2. Promastigote to Amastigote Differentiation

#### 1.2.1. Temperature and pH as Triggers

Exposure of promastigotes to elevated temperatures and acidic milieu in vitro triggers their differentiation into amastigotes in axenic culture [6,7], facilitating the analysis of key molecular events during *Leishmania* stage conversion in this direction. The trigger temperatures must be adjusted according to the tissue tropism (viscera vs. skin) of the given *Leishmania* species and strains used, e.g., 37 °C for viscerotropic L. donovani and L. infantum and 32–34 °C for cutanotropic L. mexicana and L. (V.) braziliensis. This differentiation takes ~5 days to complete and is reversible [8]. Reverse conversion of axenic amastigotes back to promastigotes takes ~2 days to complete once the incubation temperature is lowered to ~25 °C and the pH returned to 7.0–7.4, mimicking conditions inside the sand fly gut.

#### 1.2.2. Emerging Alternative/Additional Triggers

The majority of the biochemical and morphological changes seen in promastigote-to-amastigote differentiation are also observed when promastigotes are exposed to geldanamycin or radicicol, which occupy and inhibit the ATP-binding domain of the major chaperone protein, 90 kDa heat shock protein (HSP90 a.k.a. HSP83) [9,10].

There is also evidence for an involvement of iron metabolism and reactive oxygen species (ROS) in triggering the promastigote-to-amastigote conversion. Iron depletion in the growth media induces an increased *Leishmania* iron transporter 1 (LIT1) expression and stops proliferation, resulting in the appearance of an amastigote-like morphology and increased infectivity to susceptible mice [11]. This is accompanied by a higher abundance of certain amastigote-specific markers, such as amastin mRNAs. Exposure of *Leishmania* species to ROS was reported to produce a similar outcome [11,12].

#### 1.2.3. Markers of Promastigote-to-Amastigote Conversion

Apart from the morphological changes, i.e., cell rounding and the reduction of the flagellum length, detectable by microscopy, there are molecular markers to verify stage conversion, e.g., amastigote-specific A2 proteins [13,14]. They exist as a family of proteins with variable copies of 10-amino acid repeats and are associated with *Leishmania* infectivity. Their primary role, however, appears to be stress protection [15,16].

Another amastigote-specific marker was identified in a proteomics study of *L. donovani* differentiation, i.e., 2,3-trans-enoyl CoA isomerase [17]. There are two isoforms of this enzyme discernible by SDS-PAGE and Western blot analysis. Isoform 1 and isoform 2 are expressed more abundantly in promastigotes and amastigotes, respectively [8]. Nuclease P4 may be amastigote-specific [18], as much as amastins [19], but its stage-specificity is equivocal due to a lack of common standard among different laboratories to verify *Leishmania* differentiation.

### 1.3. Limitations of In Vitro Differentiation Models

While axenically grown amastigotes are a very important tool for studying the dynamics of gene expression, the axenic conditions limit their utility for other fields of research, which require exposure to the host cell environments, i.e., antimicrobial defense mechanisms, residence in parasitophorous vacuoles and nutrient supplies from infected macrophages. This is reflected in the finding that proteins that are important for intracellular survival, such as the 100 kDa heat shock protein [20] or cyclophilin 40 [21], are dispensable for axenically grown amastigotes. The metabolic pathways are reprogrammed during *Leishmania* differentiation into axenic amastigotes, as reflective of the requirements of carbon sources from mammalian versus insect hosts and also the oxidative stress encountered in the former [17,22,23].

The use of axenic amastigotes for drug screening also raises the question of their appropriateness, considering that intracellular amastigotes protect themselves not only by the barrier of their own plasma membranes and detoxifying mechanisms but also by those of the host cells and parasitophorous vacuoles. Therefore, in vitro-infected cell lines or primary host cells are more appropriate for drug testing to provide more meaningful results [24,25].

While many *Leishmania* strains of cutanotropic and viscerotropic species readily convert into axenic amastigotes, *L. major* is a notable exception. A protocol was developed for the axenization of this species [26], but it has not been widely used to assess, for example, the effects of iron depletion and exposure to ROS [11].

## 2. Transduction of Differentiation Triggers

### 2.1. Post-Transcriptional Gene Regulation during Stage Conversion

Assessing gene expression in response to triggers of differentiation begins with the understanding of *Leishmania*’s uniquely structured genome, which is organized into polycistronic transcription units (PTU), each consisting of functionally unrelated, single-copied and/or tandemly repeated genes [27]. RNA polymerase II-dependent transcription proceeds through PTUs in the absence of canonical promoters and transcription factors and therefore lacks single-gene-specific regulation [28]. Instead, our current knowledge suggests that the PTUs are constitutively transcribed, producing polycistronic pre-mRNAs [29]. These pre-mRNAs are subsequently or, likely, co-transcriptionally processed by the linked events of trans-splicing, resulting in 5′ miniexon capping and 3′ polyadenylation to produce mature, monocistronic mRNAs [30]. During conversion from promastigotes to amastigotes, *Leishmania* must rapidly change the products of their gene expression, presumably regulated primarily by post-transcriptional mechanisms, for adaptation to the changing environment [31].

*Leishmania* gene expression is modulated, at least in part, by the stability of transcripts that are available for protein synthesis. Early studies suggest that *Leishmania* mRNA stability is controlled by *cis*-elements within the 5’- and 3′ untranslated regions (UTRs). The binding of RNA-associated proteins to these regulatory RNA motifs affects not only the stability of mRNAs but also their transport and intracellular targeting [32,33]. 3’UTR *cis*-elements have been associated with the stability and abundance of HSP mRNAs, which play an important role in adaptation to the increased temperature during amastigote differentiation [34]. Stage-specific up-regulation of the amastigote-specific surface protein amastin was initially thought to be regulated via *cis-*elements in the 3′UTRs of amastin mRNAs [35] but was subsequently found to occur at the translational stage [23,36].

In general, promastigote-to-amastigote differentiation is associated with a marked overall down-regulation of gene expression, especially for transcripts involved in translation and ribosome biogenesis, which is suggestive of a regulatory role [37]. This is reflected in the lower rate of translation seen in axenically differentiated amastigotes of *L. infantum*, resulting from phosphorylation of a translation factor called eukaryotic initiation factor 2. However, the translation of amastigote-specific proteins, such as the stress protein A2, is upregulated [38]. *Leishmania* differentiation from promastigote to amastigote is associated with a switch from cap-dependent to alternative translation, involving a non-conserved 4E-interacting protein (Leish4E-IP) [39].

In addition, the processing of policystronic to monomeric mRNAs has been suggested to regulate their abundance, hence controlling *Leishmania* stage-specific differentiation [40]. RNA processing entails the *trans*-splicing of the 39-bp spliced leader (SL) RNA onto the 5’ end and the addition of a poly(A) tail to the 3’ end of all matured mRNAs. However, this early hypothesis has not been confirmed, as the processing of individual RNAs does not have a significant impact on their steady state levels. In spite of notable differences in RNA processing between procyclic and metacyclic promastigotes [41], comparative RNA-Seq and ribosome profiling analyses revealed no significant correlation between changes in mRNA levels and protein synthesis rates [23].

Furthermore, RNA synthesis rates do not appear to have any influence on the adapta- tion of *Leishmania* to changing environmental conditions during differentiation. In early nuclear run on analyses [42], heat shock was found to have no effect on HSP mRNA synthesis. We are currently investigating RNA synthesis rates during stage conversion using precision nuclear run-on/sequencing analysis (PRO-seq) [43]. Initial data analyses suggest that RNA synthesis proceeds through intergenic sequences and is specific to the positive strand of each PTU. No stage-specific changes in RNA polymerase occupancy are observed in the PTUs (J.G., unpublished data).

### 2.2. Protein Turnover and Leishmania Proteases in Stage Differentiation

*Leishmania* conversion from elongated, flagellated promastigotes into ovoid amastigotes with rudimentary flagellum requires a retooled metabolism, resulting in multiple qualitative and quantitative proteomic changes, some of which are facilitated by proteolytic events. *Leishmania* proteases are involved in three key processes of amastigote conversion: (i) controlling the abundance of stage-specific proteins; (ii) inducing apoptosis to reduce parasite loads in the host cells; and (iii) modulating the host’s immune response.

The activities of *Leishmania* peptidases are regulated by the level of their expression, modification or stability and impact on the abundance of their substrate proteins. This is reflected in the stage-specific expression of many peptidases [17,23,44,45]. Accordingly, analysis of the serine proteases LbOBP and LbS13 [46] revealed that their expression is temperature-dependent, probably via 3’UTR secondary structure motifs and PTM [47].

Both proteasomal and lysosomal pathways are involved in *Leishmania* protein degradation [48]. During metacyclogenesis, cysteine protease B (CPB) levels increase in MVT-lysosomal compartments matching their proteolytic activities [48]. Acidification induces the secretion of LbCPB by *L. braziliensis* [49], and its specific activity peaks during the first 72 h of heat exposure in vitro [50]. The CPB is then secreted via the flagellar pocket.

The development of megasomes in amastigotes is linked to their infectivity [51]. The cysteine proteases therein are crucial for the parasites’ intracellular survival [48,52]. This is most likely due to the selective degradation of MHC II surface immune complexes in the infected macrophages and cytokines in the extracellular milieu, which shifts the host’s immune response toward the Th2 pathway [53].

A broad variety of stage-dependent functions is reported for the highly abundant and stage-specifically expressed metalloproteases of the MSP family (GP63, leishmanoly- sin). Their proposed functions include: (i) making nutrients available to *Leishmania* in the sandfly vector; (ii) releasing metacyclics from adhesion to the fly midgut epithelium; (iii) neutralizing the host’s first line antimicrobial defense activities, e.g., complement-mediated lysis, anti-microbial proteins and NK-cell attacks; (iv) facilitating *Leishmania* entry into macrophages by receptor-mediated phagocytosis and their motility in the extracellular matrix; and (v) maintaining the amastigotes’ survival in the parasitophorous vacuoles [54].

*Leishmania* differentiation is accompanied by autophagic events. Stressors that induce stage differentiation also trigger apoptosis via a variety of different molecular pathways [55]. In contrast to higher eukaryotes, *Leishmania* are deficient of caspases. Instead, some leishmaniae rely on metacaspases. This was demonstrated by inducing programmed cell death (PCD) in transgenic yeast by the over expression of LmjMCA [56]. Other *Leishmania* proteases of functional importance are a large family of calpain-like proteases (CALP) and cysteine protease C (CPC) [55]. The mechanisms employed by *Leishmania* to avoid apoptosis and instead enter differentiation deserve further detailed investigation, as this has the potential to provide novel targets for specific therapeutic intervention against leishmaniasis.

### 2.3. Protein Kinases in Stage Differentiation and Intracellular Survival

With *Leishmania* lacking transcriptional control, protein modifications constitute an important aspect of regulation [57]. A kinome-wide gene deletion study revealed that 162 *L. mexicana* protein kinase genes were dispensable, while 44 genes were refractory to deletion and thus considered essential for promastigote viability. In addition, 29 kinases were found to be crucial for differentiation from the metacyclic promastigote to the amastigote and for the successful infection of macrophages in vitro and BALB/c mice [58].

These kinases are highly conserved among different trypanosomatid species (Table 1) [58,59,60,61,62,63,64,65,66,67,68,69,70,71,72,73,74,75,76,77,78,79,80,81,82,83,84]. Moreover, some essential kinases were also found to play a crucial role in stage conversion and virulence, e.g., LmxMPK4 [85] and secreted CK1.2 [86]. The complete absence of tyrosine kinases, TKL receptors and RGC (receptor guanylyl cyclases) in trypanosomatids underscores the potential significance of *Leishmania* serine-/threonine kinases and of atypical kinase [58,62,87].

*Leishmania* kinases appear to mediate a wide variety of core functions in *Leishmania* differentiation, such as heat shock and osmotic stress response, nutrient sensing and metabolic regulation, translation control, cytokinesis, motility and morphogenesis (Table 1). Interestingly, the regulatory functions of *Leishmania* kinases often differ from those of their orthologues in higher eukaryotes. This complicates the efforts to predict interaction pathways. For instance, *Leishmania* appear to lack the activation cascade of LmxMPK4 by STE kinases at the conserved TxY Motif [58,75].

Due to their apparently crucial roles, *Leishmania* protein kinases also constitute promising drug targets notwithstanding their possible side effects against the host orthologues. Yet, a recent kinome comparison revealed a parasite-host sequence similarity of 23–69% (*L. infantum* vs. *H. sapiens*) and 21–69% (*L. braziliensis* vs. *H. sapiens*) [87], raising the hope of finding inhibitors with sufficient selectivity. Re-purposing available inhibitor libraries for testing against parasite kinases may be a promising path to finding lead compounds for treating *Leishmania* infections.

### 2.4. Epigenetic Effects

The structure of eukaryotic chromatin is primarily formed by nucleosomes, consisting of genomic DNA wrapped around a core histone octamer and separated by various lengths of linker DNA. This beads-on-a-string structure or 10-nm fibre is also referred to as euchromatin and represents accessible DNA. Further condensation leads to the formation of a 30-nm fibre—heterochromatin—which is considered less accessible for transcription factors and RNA polymerases. It has been postulated that euchromatin is required for inducing gene expression under changing environmental conditions, for example, during *Leishmania* stage conversion [88].

Indeed, ribosome profiling analyses showed that the inhibition of HSP90 leads promastigotes to become amastigote-like, resulting in the elevated synthesis of histones [23]. This was also observed by proteome analyses and occurs early on in the promastigote-to-amastigote conversion [17]. In addition, histone deacetylases were found to be regulated stage-dependently, suggesting the impact of epigenetic mechanisms on stage conversion [37]. An assay for transposase-accessible chromatin by sequencing (ATAC-seq) analysis showed that axenic amastigote differentiation is associated with the emergence of heterochromatin [89] in the divergent SSR regions upstream of telomere ends where transcription is initiated [90,91]. Whether the formation of heterochromatin in upstream SSRs has an impact on transcription rates in the downstream PTUs is currently under investigation (J.G., unpublished data).

### 2.5. HSPs in Stage Conversion

Since one of the key stimuli for promastigote-to-amastigote conversion is an elevation of the ambient temperature to that of the mammalian host, we suspected early on the involvement of the heat shock response in the development of amastigotes. The expression of various heat shock proteins is upregulated when subjecting promastigotes to a temperature up-shift, independent of acidic pH [92]. Therefore, the elevated synthesis of HSPs is an early response to this differentiation stimulus alone.

Moreover, the chaperones HSP100 and HSP23, along with the putative co-chaperone cyclophilin 40, were found to be essential for *Leishmania* survival in macrophages in vitro and/or in animal hosts [93,94,95]. HSP100 is functionally important to counter the host’s immune response in the parasite’s favor by playing a pivotal role in the assembly of immune-modulatory exosomes, as shown by their ineffectiveness when produced by HSP100 null mutants. While “wild type” exosomes trigger a cytokine expression in keeping with a Th2-response and a B cell-based immune reaction, the exosomes of HSP100 null mutants fail to suppress a Th1-based inflammatory response, known to restrict *Leishmania* survival in the mammalian host. 

This is apparently due to the alteration in the composition of exosomes shed by HSP100^−/−^ mutants lacking several HSPs and virulence factors [96].

**Figure 1:**
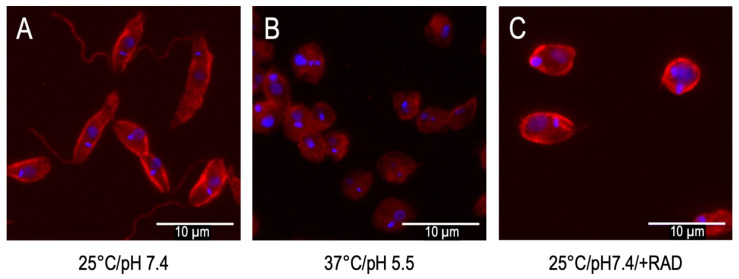
Axenic stage conversion of *L. donovani* from promastigotes kept at 25 °C/pH 7.4. (**A**) to amastigotes at 37 °C/pH 5.5 (**B**) and chemically (radicicol)-induced amastigote-like forms (**C**). Samples were fixed and stained with DAPI (nuclei, blue) and anti-α-tubulin mAB (microtubuli, red). Fluorescence images were captured at a 100× magnification using an EVOS Autofluor microscope; overlays were implemented in Adobe Photoshop CS3. The bars represent 10 µm.

The highly abundant chaperone HSP90 is involved in the transduction of differentiation signals. We first noted this when *L. donovani* promastigotes were treated with HSP90-specific inhibitors, geldanamycin or radicicol, resulting in a reduced growth and a morphological shift [9] from the long, slender, flagellated promastigotes to ovoid, non-motile amastigote-like cells, as shown in Figure 1.

Moreover, similar proteome changes were observed in axenic amastigotes and after radicicol- or 17-AAG-treatment [10]. Using ribosome profiling and BONCAT-iTRAQ mass spectrometry, respectively, the results showed elevated synthesis for a number of different proteins, including amastins, histones, fatty acid metabolic proteins, oxidative stress response proteins and several heat shock proteins, highly reminiscent of gene expression in axenic amastigotes generated by elevated temperature and acidity [23,97].

How HSP90 channels the signals to trigger *Leishmania* differentiation is unclear. It is known in yeast, plants and mammals that HSP90 and HSP70 act as negative regulators of the cellular heat shock response by quenching the activity of the HSF1 heat shock transcription factor [98]. There is little or no HSF activity detectable in homeostatic quiescent cells, but protein biosynthesis is not required for the rapid onset of HSF DNA-binding activity and heat shock gene expression [99]. Heat stress and protein folding stresses deplete the pool of free, active HSP70 and HSP90 chaperones, freeing HSF1 to form active trimers for binding its target sequences.

**Figure 2:**
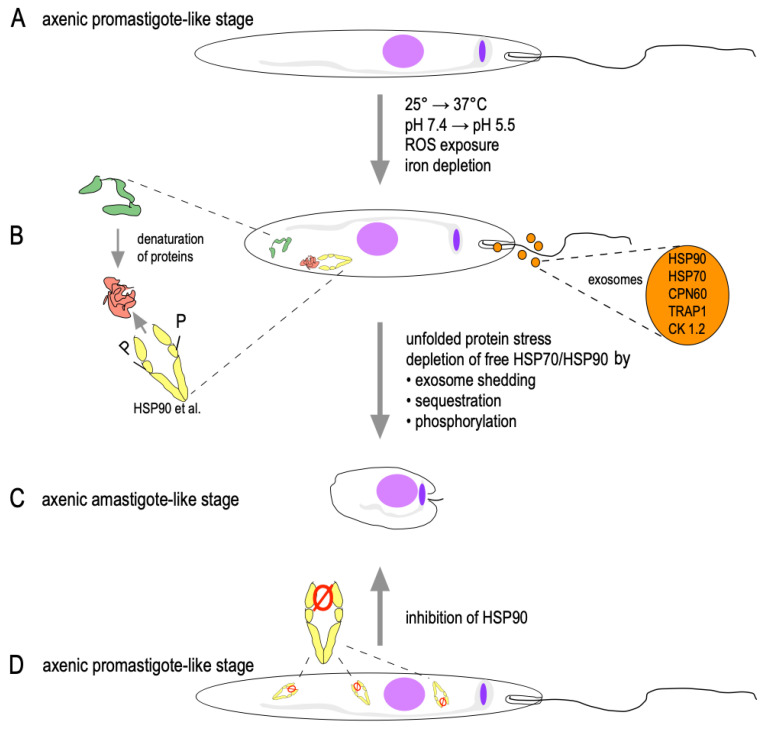
Schematic model of stage conversion through HSP90 quenching. Axenically grown promastigotes (**A**) as elongated, flagellated, highly proliferative cells are exposed to cell stress (increased temperature, acidified medium, iron depletion or ROS). This causes the sequestration of HSP90 and other foldosome components to denatured protein domains (**B**). The temperature-dependent shedding of HSP-containing exosomes and the phosphorylation of HSP90 by CK1.2 and MAPK1 adds to the reduction of free HSP90, which triggers conversion to the ovoid, non-motile, growth-impaired amastigote (**C**). Alternatively, the direct inhibition of HSP90 with radicicol or geldanamycin also depletes active HSP90 and causes a similar conversion to amastigote-like stages (**D**).

A similar depletion of HSP90 and HSP70 may occur during the exposure of *Leishmania* promastigotes to host tissue temperatures, a >∆10 °C heat shock compared with the sandfly gut. This may be enhanced by acidic milieu but also by iron depletion and ROS stress. Elevated temperature also boosts the shedding of exosomes which contain HSP90 and HSP70 as major payload proteins [100]. Such a depletion will cause HSP90-dependent regulatory proteins to be suppressed, even more so as HSP70 is also a part of the growth-promoting HSP90 multichaperone complex [101]. We speculate that HSP90 functional depletion transduces various stresses that trigger *Leishmania* stage conversion in vitro (Figure 2).

In higher eukaryotes, highly abundant HSP90 homologues are subject to phosphorylation with the concomitant modulation of its activity, but this requires the participation of protein kinases in an active or activatable state, such as MAP kinases [102,103,104]. *Leishmania* HSP90 is also the target of amastigote stage-specific phosphorylation, together with other housekeeping chaperones such as HSP70 and Sti1 [71]. HSP90 harbors several known or putative phosphorylation sites. Mutations of these sites produce a variety of different effects, ranging from the loss of cell viability to defective amastigote-specific infectivity (T223, S594, S595) to a minor reduction in proliferation [83].

So far, two protein kinases were identified that target HSP90: (i) MAP kinase 1, which was found to be essential for *L. mexicana* intracellular survival [60,81], and (ii) casein kinase 1, isoform 2 (CK1.2), which is secreted into the infected host cells and is required for intracellular survival [83,105]. The mutation of the CK1.2 phorphorylation site at S289 in HSP90 results in a slowdown of in vitro proliferation and minor morphological changes [83]. CK1.2 also interacts with HSP23 in vitro and can mediate stress resistance in the absence of HSP23 [106], while MAP kinase 1 also targets HSP70 [81].

## 3. Future Directions of Research

Recent years have seen a massive increase in experimental data about *Leishmania* stage conversion, mostly due to the application of various systems biology strategies aimed at the various levels of gene expression and their regulation. Still, there are pieces missing from the complex jigsaw puzzle on which we are working.

It is clear by now that mRNA abundance is relevant to, but not a reliable measure of, *Leishmania* gene expression. Since *Leishmania,* like all other trypanosomatids, regulate gene expression post-transcriptionally, it is more promising to direct our attention to the mechanisms of RNA utilization by ribosomes and translation factors. Still, a systems biology approach to correlate RNA stability with RNA abundance is expected to help explain the fluctuations of mRNA steady state levels observed in transcriptome analyses. *Leishmania* stage-specific gene expression is regulated primarily at the level of translational efficiency via the interactions of RNA-binding proteins with mRNAs for their stability and effective processing.

Proteome and translation analyses have shown that the expression patterns of individual ribosomal proteins undergo changes, suggestive of alterations in the composition and specificity of ribosomes during *Leishmania* stage conversion. Specific stress ribosomes have been reported to form under environmentally challenging conditions, leading to the preferential translation of specific mRNAs in bacteria. Post-translational protein modifications mediated chiefly by the activities of protein kinases may regulate the activity and specificity of translation initiation and elongation, signifying the need for investigation in this area and in the interactions between protein kinases and chaperones. It is essential to standardize the inducers of *Leishmania* stage conversion in vitro for examining the downstream pathways at the translatome and (phospho-) proteome levels to develop a unified model of signal transduction regulating this cyclic differentiation. The outcome of such investigation with the in vitro axenic differentiation model requires verification in vivo, that is now possible with the progress in single-cell sequencing, to study intracellular amastigotes and sand fly gut-derived promastigotes by NGS-based gene expression assays.

## Figures and Tables

**Table 1:** *Leishmania* protein kinases and their known roles/functions in stage differentiation.

Family	Kinase	Reported Function	Reference
CMGC/CDK	Lmx/T.br/Tc/LmjCRK7	No functional homologue to human CDK7	Baker et al.,2021 [53] Badjatia et al., 2013 [70] Parsons et al., 2005 [58]
	LmxCRK8	Untypical regulation indicated in TrCRK8	Baker et al., 2021 [53] Hammarton 2007 [60]
CMGC/CDKL and CMGC/MAPK	Lmx/LdMPK1	Intracellular survival, phosphorylating LdHSP70 and LdHSP90; antimony resistance	Baker et al., 2021 [53] Wiese, 1998 [56] Hombach-Barrigah et al., 2019 [79] Kaur et al., 2017 [77] Morales et al., 2010b [67] Garg and Goyal, 2015 [72]
	LmxMPK2	Infection; essential nutrient regulation and osmotic stress via Arginine depletion response (ADR) and AQP1-regulation; antimony resistance flagella-mediated environment sensing	Goldman-Pinkovich et al., 2016 [75] Kelly et al., 2021 [80] Mandal et al., 2012 [69] Rotureau et al. 2009 [64]
	LmxMPK15	Infection	Baker et al., 2021 [53]
	Lmx/LmjMPK10	Stage-specific auto-regulation and phosphorylation crucial for infection; crystal structure available; not crucial in *L. major*	Morales et al., 2007 [62] Horjales et al., 2012 [68] Cayla et al., 2014 [71]
	LmjMPK7	Infection	Morales et al., 2010 [67]
STE	Lmx/Lmj/T.brMRK1	Cytoplasmatic MAP3K; infection; osmotic challenge in *T.brucei*	Baker et al., 2021 [58] Agron et al., 2005 [57] Fernandez-Cortes et al., 2017 [76]
CK1.2	LdCK1.2	Exosomal kinase; phosphorylates HSP90 and HSP23	Hombach-Barrigah et al., 2018 [79] Kröber-Boncardo et al., 2020 [87]
Other/CK2	LmxCK2A1, LmxCK2A2	T.brCK2 linked to cytoskeletal processes; LbrCK2 secreted and ekto-forms mediating virulence	De Lima et al., 2006 [59] Zylbersztejn et al., 2015 [74] Dutra et al., 2009 [63]
PEK	LmxEIF2αK2	Vital for infection; T.brEIF2αK2 linked to sensing or transport	Baker et al., 2021 [53] Moraes et al., 2007 [61]
PIKK related	LmxTOR3	Infection; acidocalcisome formation and metabolic regulation	Baker et al., 2021 [53] Madeira da Silva and Beverley, 2010 [65]
CAMK	LmxAKB1	Infection; T.brAKB1: cytokinesis and division	Inoue et al., 2015 [73]
AGC/PKA	LmxPKAC3	Infection; morphogenesis	Fischer Weinberger et al., under review [53]
Other/ULK	LmxSTK36, LmxULK4	Infection of sandfly vector and mammal. functionally linked. T.brSTK36, Tbr.ULK4: motility and flagella assembly	Baker et al., 2021 [51] Varga et al., 2017 [76]

## Data Availability

Not applicable.
